# Reprogramming of HUVECs into Induced Pluripotent Stem Cells (HiPSCs), Generation and Characterization of HiPSC-Derived Neurons and Astrocytes

**DOI:** 10.1371/journal.pone.0119617

**Published:** 2015-03-19

**Authors:** Yohannes Haile, Maryam Nakhaei-Nejad, Paul A. Boakye, Glen Baker, Peter A. Smith, Allan G. Murray, Fabrizio Giuliani, Nadia Jahroudi

**Affiliations:** 1 Department of Medicine, University of Alberta, Edmonton, Canada; 2 Department of Pharmacology, University of Alberta, Edmonton, Canada; 3 Neuroscience and Mental Health Institute, University of Alberta, Edmonton, Canada; 4 Department of Psychiatry (Neurochemical Research Unit), University of Alberta, Edmonton, Canada; University of Düsseldorf, GERMANY

## Abstract

Neurodegenerative diseases are characterized by chronic and progressive structural or functional loss of neurons. Limitations related to the animal models of these human diseases have impeded the development of effective drugs. This emphasizes the need to establish disease models using human-derived cells. The discovery of induced pluripotent stem cell (iPSC) technology has provided novel opportunities in disease modeling, drug development, screening, and the potential for “patient-matched” cellular therapies in neurodegenerative diseases. In this study, with the objective of establishing reliable tools to study neurodegenerative diseases, we reprogrammed human umbilical vein endothelial cells (HUVECs) into iPSCs (HiPSCs). Using a novel and direct approach, HiPSCs were differentiated into cells of central nervous system (CNS) lineage, including neuronal, astrocyte and glial cells, with high efficiency. HiPSCs expressed embryonic genes such as nanog, sox2 and Oct-3/4, and formed embryoid bodies that expressed markers of the 3 germ layers. Expression of endothelial-specific genes was not detected in HiPSCs at RNA or protein levels. HiPSC-derived neurons possess similar morphology but significantly longer neurites compared to primary human fetal neurons. These stem cell-derived neurons are susceptible to inflammatory cell-mediated neuronal injury. HiPSC-derived neurons express various amino acids that are important for normal function in the CNS. They have functional receptors for a variety of neurotransmitters such as glutamate and acetylcholine. HiPSC-derived astrocytes respond to ATP and acetylcholine by elevating cytosolic Ca^2+^ concentrations. In summary, this study presents a novel technique to generate differentiated and functional HiPSC-derived neurons and astrocytes. These cells are appropriate tools for studying the development of the nervous system, the pathophysiology of various neurodegenerative diseases and the development of potential drugs for their treatments.

## Introduction

Neuronal loss is the hallmark of neurodegenerative diseases such as multiple sclerosis (MS), amyotrophic lateral sclerosis, Parkinson’s-, Alzheimer’s-, and Huntington’s diseases. It is widely reported that genetic mutations and environmental factors contribute to the pathogenesis of these diseases [1–3]. However, the goal of developing effective therapies for these diseases has not yet been achieved. A major hindrance towards this goal is the lack of appropriate models. Limitations of animal models accurately mimicking human pathophysiology are confounding factors in the failures of many potential drugs [4]. This emphasizes the need for disease models that are based on human cells [5–7].

The landmark report of generation of induced pluripotent stem cells (iPSCs) [8,9] from somatic cells has opened new avenues (without ethical concerns and immune rejection) in modeling various human diseases, drug screening/discovery, transplantation in animal models and regenerative medicine [10–12]. Human iPSC-derived neuronal cell models offer unrestricted access to early stages of disease pathogenesis [13].

iPSCs and their differentiated progenies, including neurons, have been generated from various cell sources, with variable kinetics and efficiencies. Nevertheless, harvesting somatic cells to establish human iPSCs should pursue non/minimally invasive procedures and minimize any possible associated risks to the donor. Dermal fibroblasts, from which the first human iPSCs were developed [9], are commonly used. However, disease modeling and development of therapeutic applications of adult skin-derived iPSCs may be limited because of accumulated mutations resulting from aging and UV exposure [14]. Alternatively, human umbilical vein endothelial cells (HUVECs) are an attractive somatic cells source for therapeutic-grade iPSCs due to their accessibility without invasive methods, availability, donor cell age, high efficiency of isolation and proliferation, as well as rapid kinetics of reprogramming [14,15]. These fetal cells have no/less environmental or technically induced DNA damage and are likely to have acquired fewer genetic mutations compared to adult-derived somatic cells [4,16]. Furthermore, HUVECs express high levels of endogenous KLF4 [17], suggesting ease of reprogramming. All these features make HUVEC-derived iPSCs an ideal cell source for developing disease models, testing therapies, or using as controls for patient-derived iPSCs within a family when investigating genetically heritable diseases [18,19].

In this study, we planned to develop a reliable tool with which to study neurodegenerative diseases. We generated iPSCs from HUVECs (HiPSCs) without the use of a feeder layer, which is a crucial step for advancing iPSC research to human therapeutic applications [20]. Using a novel approach, we differentiated HiPSCs into mature and functional neurons and astrocytes with a significantly high efficiency. We established a direct differentiation protocol without the use of embryoid bodies. Direct differentiation approaches are more convenient, require fewer reagents, and may be more consistent in terms of efficiency and generation of higher yields of desired cell types [21,22]. We assessed and characterized the morphology, susceptibility to inflammatory cells and amino acid contents, as well as functionality of the receptors to various stimulants (such as glutamate, nicotine, ATP and acetylcholine) of HiPSC-derived neurons and astrocytes in comparison to primary human fetal cell-derived neurons and astrocytes. In summary, using a novel technique, this study developed a tool that can serve as an appropriate cell model for the study of a variety of neurodegenerative diseases.

## Materials and Methods

### Ethics Statement

The University of Alberta Biomedical Ethics Committee (UABEC) approved the protocols for human umbilical cord collection and isolation of human umbilical vein endothelial cells (HUVECs) [23], for collection of human brain tissue from therapeutic abortions of 15–20 week fetuses and isolation of primary cells, as well as collection of blood samples from healthy adult volunteer donors for cell isolation. The procedures were performed in agreement with the guidelines approved by UABEC, the principles outlined in the Declaration of Helsinki and also Title 45, US Code of Federal Regulations, Part 46, Protection of Human Subjects, effective December 13, 2001. The researchers also followed the recommendations of the Royal Commission on New Reproductive Technologies (http://www.pre.ethics.gc.ca/eng/archives/tcps-eptc/section9-chapitre9/#9D). For human brain tissue and umbilical cord, the donors’ mothers provided informed consent in writing before donating the tissues. For collection of blood samples and isolation of human peripheral blood mononuclear cells (HPBMCs), the donors provided informed verbal consent and their names were documented in a blood donor registry prior to inclusion in the study. All consent procedures were approved by the local ethics committee, and all the experiments were conducted within the University of Alberta.

### Reagents

M199, MEM medium, RPMI1640, AIM-V T cell culture medium, FBS, glutamine, penicillin/streptomycin, ECGS, both B27 with insulin and without insulin, essential amino acids, sodium pyruvate, and dextrose were purchased from Invitrogen, Life Technologies (Burlington, ON). Matrigel hESC-qualified Matrix was from BD Biosciences, ON. Lentiviral vectors were from viPS Vector Kit (ThermoFisher Scientific, catalogue number IPS5449). Activin A was from R&D Systems (Minneapolis, MN). mTeSR1 medium, Accutase, Aggrewell plates, Aggrewell medium, Rock inhibitor (Y-27632), and DMEM/F12 medium were from Stem Cell Technologies Inc, BC; RNeasy Plus mini kit was from Qiagen, Ontario, and qScript synthesis kit was purchased from Quanta Biosciences, VWR international.

### Reprogramming of HUVECs into induced pluripotent stem cells (iPSCs)

HUVECs were a gift from Dr. Sandra Davidge (University of Alberta). HUVECs were prepared and maintained in culture as previously described [23]. To generate iPSCs without the use of mouse embryonic feeder layer, HUVECs (passages 2–3) were seeded on BD Matrigel hESC-qualified Matrix that had been optimized to support iPSC growth for up to 25 passages [24,25]. Twenty-four hours (h) after seeding, cells were approximately 70% confluent, and were transduced with 5MOI of six lentiviral vectors expressing the genes Lin28, c-Myc, Klf4, Nanog, Sox2 and Oct4. In parallel, HUVECs were transduced with 5MOI GFP-lentivirus to assess the transduction efficiency. HUVECs were maintained in complete endothelial cell growth medium (M199, 20% FBS, 2 mM glutamine, 100 IU/ml penicillin/streptomycin and 100 μg/ml ECGS) for 4 days, which was then replaced with mTeSR1 medium. The medium was changed daily after this point. After 2 weeks, colonies with morphologic characteristics of pluripotent stem cells (round colonies with defined edges, individual cells within the colony had a high nuclear to cytoplasmic ratio, and were tightly packed with defined junctions) started to emerge. After 3 weeks, desirable colonies were selected and passaged on Matrigel according to the protocol described by the Stem Cell Technologies mTeSR1 manual.

### RNA preparation and RT-PCR

Total RNA was isolated from cells and purified and genomic DNA was removed using an RNeasy Plus mini kit. RNA was reverse transcribed into cDNA using qScript synthesis kit and subjected to real time PCR (RT-PCR) analysis using Fast 7500 thermocycler (Applied Biosytems). The primers used for detection of various mRNA abundance are listed in table 1. Targeted mRNA values were normalized to HPRT.

**Table 1:** List of primers and their sequences or catalogue numbers used in the study.

Primer	Forward	Reverse
Nanog	CAGTCTGGACACTGGCTGAA	CTCGCTGATTAGGCTCCAAC
SOX2	CAAAAATGGCCATGCAGGTT	AGTTGGGATCGAACAAAAGCTATT
Oct4	GGGTTTTTGGGATTAAGTTCTTCA	GCCCCCACCCTTTGTGTT
Pax6	Qiagen: QT00071169	Qiagen: QT00071169
VEGFR2	GGAACCTCACTATCCGCAGAG	CCAAGTTCGTCTTTTCCTGGGC
VWF	Qiagen: QT00051975	Qiagen: QT00051975
CD31 (PECAM1)	Qiagen: QT00081172	Qiagen: QT00081172
Tie-2	Qiagen: QT01666322	Qiagen: QT01666322
eNOS	GCCACCGCCGTGAAGATCT	CATACAGGATTGTCGCCTTCA
VE-cadherin	Qiagen: QT00013244	Qiagen: QT00013244

### Generation of embryoid bodies (EB)

Reprogrammed HUVECs were treated with Accutase to generate a single cell population, then were placed in Aggrewell plates with Aggrewell medium containing 10 μM Rho kinase inhibitor (Y-27632) for 24 h. Embryoid bodies generated by this method were transferred to non-tissue culture plates in DMEM/F12 medium containing knockout serum for 8 days. RNA from EB was isolated as described above. Upregulation of markers specific to each germ layer was tested by either RT-PCR or immunofluorescence microscopy.

### Differentiation of HiPSCs into neurons and glial cells

Directed differentiation of HiPSCs followed the protocol by Laflamme et al [21] with modifications as follows. HiPSCs were seeded on a matrigel-coated T-25 flask or 6-well plates supplemented with mTeSR1. After 6–7 days, when cells reached 70% confluency, direct differentiation was initiated by changing the medium to RPMI1640 supplemented with 100 ng/ml Activin A and 1xB27 without insulin for 24 h. Then, fresh medium containing the above supplements and 10 ng/ml BMP4 (bone morphogenetic protein-4) was added to the cells for the next 4 days, with medium change every two days. Subsequently, the medium was changed to RPMI1640 + B27 supplemented with insulin in the presence of 0.5% penicillin/streptomycin, and exchanged every two days. The cell culture was incubated at 37°C with 5% CO_2_ for 2–3 weeks. Neurons and astroglia with distinct morphologies appeared after 12 days.

### Human fetal neurons isolation and culture

Human cortical fetal neurons (HFNs) were isolated from brain tissues and processed as previously described [26]. Enriched neuronal cells were trypsinized and plated (500,000 cells/well) onto 24-well cell culture plates (Nunc, Naperville, IL) for 3 days before co-culturing with T cells (for neuronal killing assay). The maturity of HFNs was previously reported based on the expression of neuron-specific biochemical markers, morphological differentiation, and physiological properties of the neurons [27].

### Culture of human T cells

Human peripheral blood mononuclear cells (PBMCs) were isolated from blood samples of healthy adult volunteer donors using Ficoll-Hypaque centrifugation separation, and suspended in serum-free AIM-V T cell culture medium. T cells were plated at a density of 200,000 cells/well in 200 μl media, and maintained for 3 days on multi-well culture plates that were either untreated (for unactivated control cells) or coated with 5 μg/ml human anti-CD3 antibody (for induction of T cell activation).

### Gene Profiling

RNA was isolated using an RNeasy Plus mini kit (Qiagen) and checked for integrity using an Agilent bioanalyser. RNA was amplified and hybridized to the Affymetrix Primeview Chip according to the manufacturer’s protocol (Affymetrix, Santa Clara, CA). Amplifications and microarray were performed by the Alberta Transplant Applied Genomics Centre (ATAGC, University of Alberta, AB). Analysis was done using GeneSpring software, Agilent Technologies. hESCs (H9 human embryonic stem cells, GEO accession# GSE48257), hESC-SCNTs (hESCs generated by somatic cell nuclear transfer GEO accession# GSE46397) and hESC-NSCs (hESCs differentiated into neuronal stem cells, GEO accession# GSE48257) that were previously analyzed by an Affymetrix Primeview Chip were used for comparison.

### Flow cytometry

HFNs or HiPSC derived cells were cultured as described above. Flow cytometry was conducted according to the manufacturer’s instructions (BD Stemflow, BD Biosciences, Mississauga, ON). Briefly, cells were detached from matrix by Accutase treatment. Cells in suspension were fixed, permeabilized and incubated with the indicated conjugated antibodies, using reagents and instructions provided by the manufacturer.

### Killing assay

HFNs (500,000 cells/well) were cultured on poly-ornithine-coated 24-well culture plates for 3 days. In parallel, T cells were activated with anti-CD3 (5 μg/ml)-coated multi-wells. After 3 days, activated T cells were co-cultured with HFNs in a 1:1 ratio. The control neuronal culture groups were treated with only AIM-V medium (without T cells) or co-cultured with unactivated T cells. After 24 h of co-culture, the wells were washed and the neurons were stained by immunocytochemistry.

### Immunocyto/histochemistry

For immuno-staining, cells were fixed with 4% PFA and permeabilized with 0.1% Triton X-100. Non-specific binding was blocked with 5% goat serum in PBS. The following antibodies were applied on each well independently: mouse monoclonal primary antibody against human microtubule associated protein-2 (MAP-2) or against human βIII-tubulin, rabbit anti-human tyrosine hydroxylase (a gift from Dr. Yves Sauve, University of Alberta), rabbit anti-glial fibrillary acidic protein (GFAP, 1:500), rabbit monoclonal against S100B (1:100; abcam), mouse anti-SSEA-4 (10 μg/ml; Millipore), mouse anti-human CD31 (1 μg/ml; BD Pharmingen), rabbit anti-VE-cadherin (4 μg/ml; Cayman, Cedarlane laboratories) and mouse anti-alpha-fetoprotein (1 μg/ml; abcam). After incubation with either Alexa 594- or 488- conjugated secondary antibodies the coverslips were mounted with ProLong Gold antifade reagent with DAPI (Invitrogen, molecular probes), and visualized under fluorescence microscopy.

### High-Performance Liquid Chromatography (HPLC)

HPLC analysis was conducted using a method slightly modified from the procedure in Grant et al. [28]. Cell pellets were prepared from the neuronal culture of HiPSC-derived neurons or HFNs. Samples were centrifuged at 12,000 x g for 5 minutes at 4°C and any residual culture media was removed. Each sample was homogenized in 100 μl of ice-cold Millipore filtered water. Ten μl of supernatant were removed and added to 40 μl of ice-cold methanol. The mixture was vortexed, left for 10 minutes on ice and then centrifuged at 12,000 x g for 5 minutes at 4°C. A 5 μl aliquot of the supernatant and 5 μl of derivatizing reagent (2 mg N-isobutyryl-L-cysteine, 1 mg o-phthaldialdehyde dissolved in 0.1 ml methanol followed by addition of 0.9 ml 0.1 M sodium borate buffer) were mixed and the mixture held in the injection loop for 5 minutes prior to injection onto a Waters 2695 Alliance HPLC. Separation was carried out on a Symmetry C18 column (4.6 mm x 150 mm x 3.5 μm) (Waters) coupled with a guard column containing the same stationary phase. The column heater was set at 30°C and the sample cooler was held at 4°C. The flow rate was 0.5 ml/min with a gradient to provide adequate separation. Mobile phase A consisted of 850 ml of 0.04 M sodium phosphate buffer and 150 ml methanol, pH 6.2. Mobile phase B consisted of 670 ml of 0.04 M sodium phosphate buffer, 555 ml methanol and 30 ml tetrahydrofuran, pH 6.2.

Initial conditions: 83% A, 17% B at 0.5 ml/min; Final conditions: 100% B at approximately 45 min. Run time: 60 minutes for column washout and equilibrium. All compounds eluted in 30 minutes. A Waters 475 fluorescence detector with an excitation wavelength of 344 nm and emission wavelength of 433 nm was employed in this assay. Proteins were quantitated with a BCA protein assay kit (Fisher) and the concentration of amino acids was reported per mg of protein.

### Confocal calcium imaging

Dynamic changes of free cytosolic Ca^2+^ concentration were monitored with confocal microscopy as previously described [29,30]. Briefly, a mixed culture of HiPSC-derived neurons and astrocytes as well as mixture of HFNs and human fetal astrocytes (HFAs) were plated on a 10 mm cover slip inserted in a 24-well culture plate and incubated at 37°C for 3–5 days supported with their respective media (described above). For Ca^2+^ imaging, superfusate containing (in mM) 130 NaCl, 4 KCl, 1 MgCl_2_, 2 CaCl_2_, 10 HEPES, and 10 glucose (pH 7.35) was applied at a rate of 3 ml/min using a roller pump (Watson-Marlow Alitea, Sin-Can, Calgary, AB, Canada). Cells were incubated with the membrane-permeant acetoxymethyl form of the fluorescent Ca^2+^-sensitive dye Fluo-4-AM (5 mM Invitrogen, Canada) for 1hr prior to imaging. Fluorescence intensity was monitored with a FV-300 laser-scanning confocal microscope (Olympus FV300, Markham, Ontario, Canada) equipped with an argon laser (488 nm) and excitation/emission filters (FF02–520/28–25; Semrock Inc, New York, NY) for emission wavelength at 514 nm, measured with a NA 0.95 20x XLUMPlanF1 objective (Olympus). Images were acquired at scan rates of 1.25–1.43 per second using a 2–3x digital zoom at full frame (512 × 512 pixel) resolution. The following drugs and their concentrations were used: ATP (100 μM), glutamate (100 μM), nicotine (100 μM) and acetylcholine (100 μM). Drugs were applied for 1 minute. Regions of interests (ROIs) were drawn around distinct cell bodies (or areas of the soma of HiPSC-derived astrocytes/HFAs that were not covered by neuronal processes, and bigger in size) and analysis of time courses of changes in fluorescence intensity was done with FluoView v.4.3 software (Olympus). ROIs of 14–15 cells/group or per culture dish, maximum of 2 dishes from each batch of cell preparation and a total of 3 batches of cells were analyzed.

The presence of HFAs in HFNs or astrocytes in the HiPSC-derived neuronal culture was easily distinguishable based on the morphology (shape and size) of the cells. The neurons possess small round or bipolar soma 15–25 μm in diameter and long processes whereas the astrocytes have irregularly shaped flat cell body and a size of more than 100 μm. Moreover, the astrocytes were immunocytochemically characterized using the astrocyte markers anti-GFAP and anti-S100B antibodies.

### Statistical analysis

Results were statistically analyzed using GraphPad Prism 5 and presented as mean ± SEM. The groups were compared using one-factor analysis of variance (ANOVA) followed by the Tukey post hoc test for normally distributed data. A two-tail unpaired t-test was applied to compare two groups with normally distributed data. P-values of <0.05 were considered significant. Asterisks represent *P<0.05, **P<0.01 and ***P<0.001.

## Results

### Reprogramming HUVECs into induced pluripotent stem cells (iPSCs)

**Fig 1:**
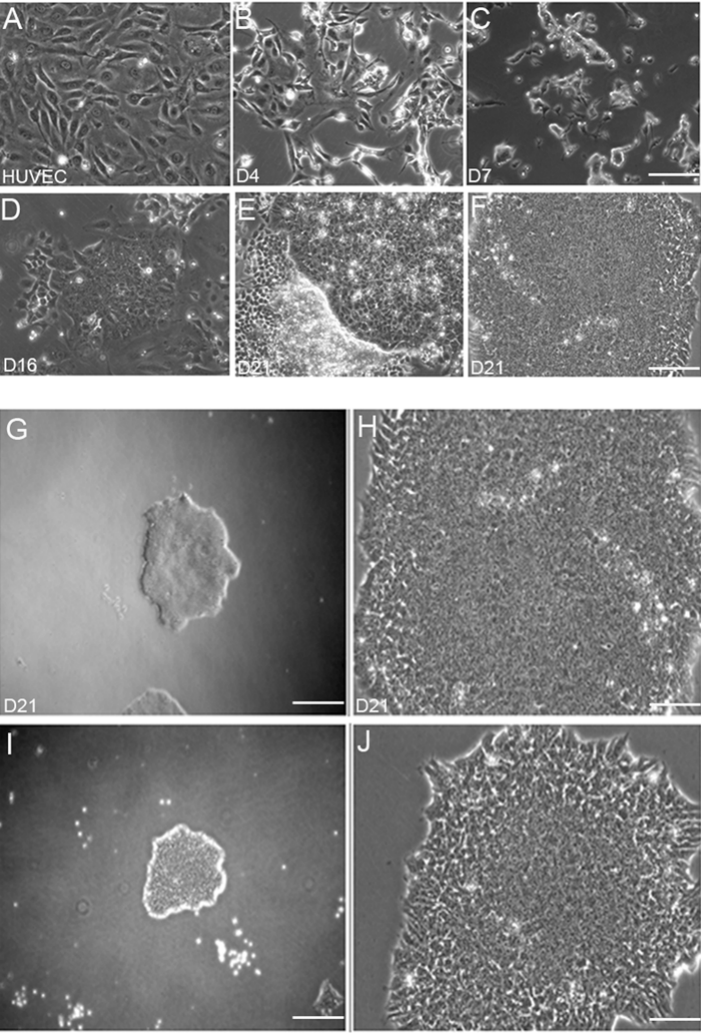
Feeder-layer independent induced pluripotent stem cells generation from HUVECs (HiPSCs). (A) HUVECs were transduced with lentiviral vectors. Within 4 days (B) and a week (C) the endothelial cells were reduced in density but formed aggregates. (D) On day 16, immature colonies emerged. (E and F) Fully reprogrammed human embryonic stem cells (hESC)-like colonies were isolated after 21 days. Micrographs (G and I in low magnification) and (H and J in high magnification) show the morphology and quality of the colonies. Scale bars: A-F = 200 μm, G and I = 100 μm, H and J = 400 μm.

HUVEC-derived iPSCs (HiPSCs), using feeder-layers, have been established and are well characterized [14–16]. Since contamination of iPSCs with animal cells is a major limitation for potential therapeutic purposes, we aimed to generate iPSCs from HUVECs independent of feeder layers. Using the protocol described in the Materials and Methods, HUVECs were transduced with lentiviral vectors expressing reprogramming transcription factors. Transduction efficiency was 78–80%, determined based on GFP expression by cells that were independently transduced with a lentivirus vector expressing GFP. Four days post transduction, the HUVEC monolayer (Fig. 1A) started to reduce density (Fig. 1B). Within a week, the density of the cells was reduced and aggregated cells started to form (Fig. 1C). On day 16, colonies emerged (Fig. 1D), and on day 20, fully reprogrammed colonies were formed (Fig. 1E and F). The morphology of the colonies was typical for iPSCs [31] and showed a well defined edge composed of tightly packed round and uniformly sized cells (Fig. 1G, I in low magnification; and H, J in high magnification).

**Fig 2:**
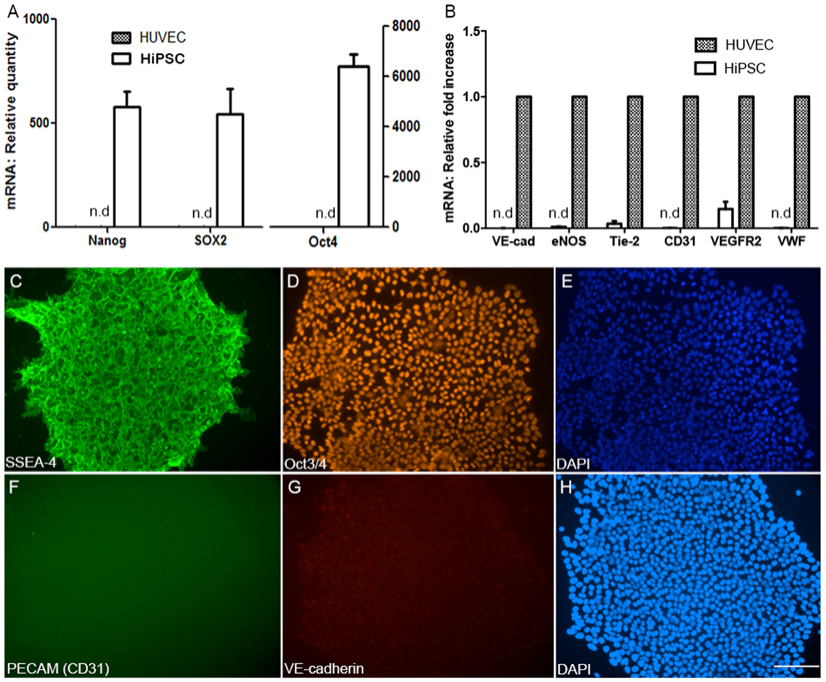
Characterization of HUVEC-derived iPSCs (HiPSCs). Gene expression was determined after 5 passages of the colonies. RT-PCR results (mRNA levels) showing endogenous embryonic (A) and endothelial (B) specific genes expressed by HUVECs and HiPSCs. Values in Figure B represent fold reduction compared to HUVECs. Data are average values of 4 HiPSC lines (N = at least 3 replicates; P<0.001). Non-detectable genes are denoted as n.d. At protein levels using immune-staining, representative micrographs show expression of embryonic markers-SSEA-4 and Oct 3/4 (C and D respectively), DAPI (E) and lack of expression of endothelial markers- PECAM (CD31) and VE-cadherin (F and G respectively) in HiPSC colonies. H is DAPI co-staining of panels F and G. Scale bar: 200 μm.

To determine if the somatic endothelial cells were efficiently reprogrammed into HiPSCs, total RNA was isolated from HUVECs at Day 0 before reprogramming, and after mature colonies were formed, then the expression of embryonic- and endothelial-specific genes were assessed using quantitative RT-PCR analyses. The embryonic primers were specifically designed to recognize endogenous genes (Table 1). The results demonstrated that the reprogrammed cells (HiPSCs) express embryonic/pluripotency-related genes such as Nanog, Sox2 and Pou51 (Oct3/4) whereas the parental HUVECs did not express embryonic genes (Fig. 2A; P<0.001). Moreover, HiPSCs did not express endothelial cell-specific genes such as VE-cadherin, eNOS, Tie-2, CD31, VEGFR2 and VWF (Fig. 2B; P<0.001), which were expressed by HUVECs, suggesting that the cells did not retain the endothelial identity. We further characterized the expression of pluripotent and endothelial markers at the protein levels using immunocytochemistry. Immuno-staining revealed that the HiPSC colonies express pluripotency markers such as SSEA-4 and Oct 3/4 (Fig. 2C and D respectively), but not endothelial markers PECAM and VE-cadherin (Fig. 2F and G respectively). Panel E in Fig. 2 is DAPI co-staining for panel D whereas panel H is for panels F and G.

**Fig 3:**
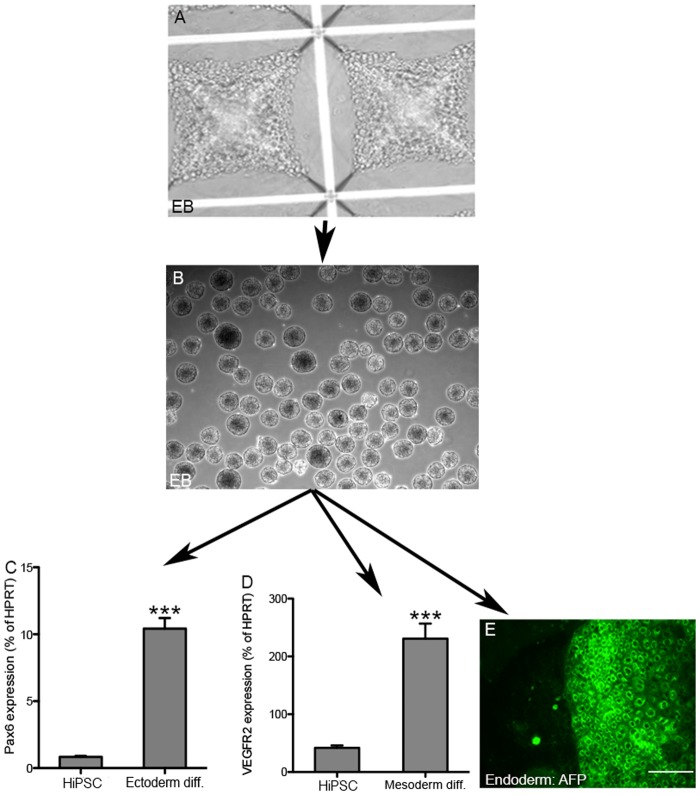
Differentiation of HiPSCs to embryoid bodies (EB) and germ layers. The ability of HiPSCs to form EB was demonstrated by dispersing colonies to single cell suspensions and placing them in aggrewell plates (A). After 24 hours, homogenous EB were formed (B). EB were differentiated to three germ layers as described in the Materials and Methods. (C and D) Germ layer formation was demonstrated by RT-PCR (C, Pax6 for ectoderm, and D, VEGFR2 for mesoderm markers respectively). (E) The endoderm marker, alpha-fetoprotein (AFP), was detected by immunofluorescence. Scale bar: 200 μm. The experiments in panels C and D were repeated 3 times; P<0.001.

Then, to characterize HiPSC pluripotency, we tested the ability of the HiPSC colonies to form embryoid bodies (EB) and differentiate to various germ layers. Colonies were lifted and turned into single cell suspensions, then aggregated in conical wells (Fig. 3A). After 24 h, the cells organized into EB structures (Fig. 3B). Expression of markers for three germ layers, i.e Pax6 for ectoderm, VEGFR2 for mesoderm and alpha-fetoprotein for endoderm were demonstrated by either RT-PCR or immunocytochemistry (Fig. 3C-E respectively). Altogether, the morphology, gene expression and differentiation capacities of HiPSCs indicate that HUVECs were successfully reprogrammed into pluripotent cells.

### HiPSCs generate mature neurons and astroglia

**Fig 4:**
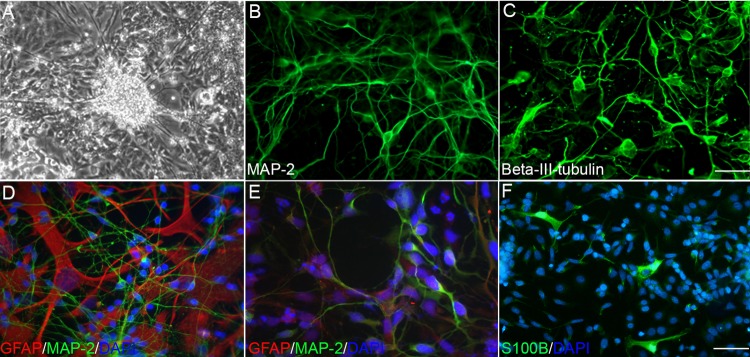
Differentiation of HiPSCs into neurons and astrocytes. Representative micrographs showing that differentiated HiPSCs acquired neuronal morphology with extending neuritic processes (A) in phase contrast, and became positive for (B) MAP-2 and (C) βIII-tubulin. (D) Primary human fetal cells differentiate into neurons (green) and GFAP-positive astrocytes (red). Similarly, HiPSCs differentiated not only to neurons but also to GFAP-positive astrocytes. The micrograph in (E) shows a HiPSC-derived mixed culture of neurons (green) and GFAP immune-stained astrocytes (red). (F) A micrograph showing few mature astrocytes positive for S100B. Blue is DAPI for nuclear staining. Scale bar: 200 μm.

The expression of ectodermal markers after the formation of 3-dimensional EB indicated that HiPSCs might differentiate into ectoderm derivatives such as neurons and astrocytes. Neuronal and astrocytic markers were expressed, as indicators of ectodermal germ layer lineages, following an indirect differentiation approach in EB [14]. In this study, we developed and pursued a direct differentiation protocol without the use of EB. As described in the Materials and Methods, sequential treatment of cells with Activin A and then Activin A plus BMP4 in the presence of neuronal growth factor B27 led to formation of neuronal cells. Many cells acquired a neuronal appearance with spindle shape cell bodies and neuritic outgrowths (Fig. 4A., phase contrast). Differentiated HiPSC-derived cells with neuronal morphology (HiPSC-Ns) expressed human neuronal markers, MAP-2 and βIII-tubulin (Fig. 4B and C respectively), suggesting that they were differentiated into immature and mature neurons.

Primary fetal brain cells differentiate to neurons and glial cells. By inhibiting the mitotic activities of astrocytes, pure primary human neuronal cultures can be generated from fetal brain cells [26,32,33], thus minimizing the dose of mitotic inhibitor is expected to increase the number of astrocytes. In culture conditions with the absence or low concentration of Ara-C (Cytosine β-D-arabinofuranoside), we demonstrated generation of an astrocyte-dominated mixed culture of MAP-2 (green) positive primary neurons and GFAP (red) positive primary human astrocytes (Fig. 4D) from fetal brain cells. Similarly, HiPSCs differentiated into both neurons and astrocytes (Fig. 4E). In addition, few cells were positive for S100 calcium binding protein B (S100B), which is a glial-specific protein and is primarily expressed by mature astrocytes (Fig. 4F). In both HiPSCs and fetal brain-derived cells, the GFAP/S100B-positive astrocytes are morphologically larger and flat, unlike the small, spindle-shaped cell body of the neurons. Quantification of MAP-2 positive neurons in relation to total cells as determined by DAPI (blue) staining of nuclei revealed that the differentiation efficiency of the neurons is close to 60% (57 ± 2.8). This high differentiation efficiency was achieved without cell sorting or enrichment.

### Microarray-based gene analysis

**Fig 5:**
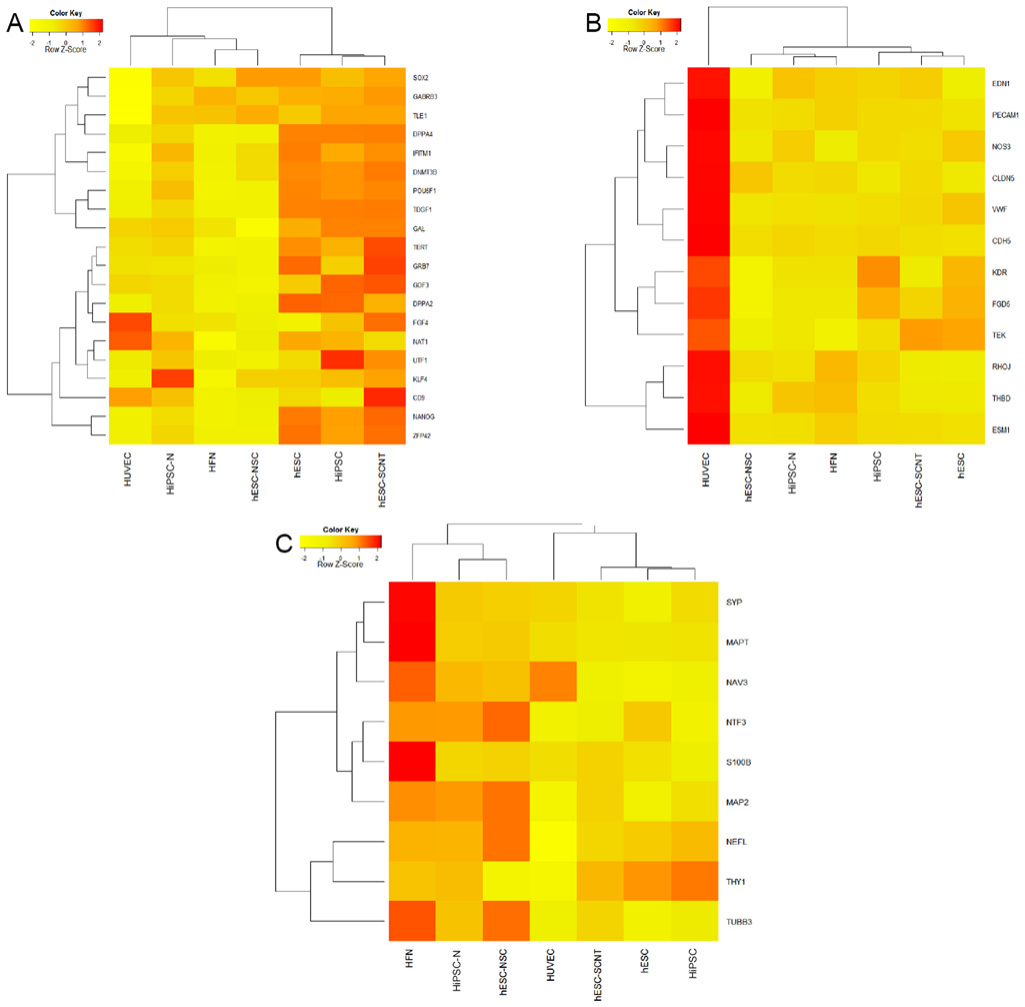
Microarray-based gene analysis. The graphs show cluster analysis of Row Z-score data of (A) pluripotent/embryonic, (B) endothelial, as well as (C) neuronal and glial genes expressed by HUVECs, HiPSCs, HiPSC-derived neurons (HiPSC-Ns), HFNs and samples obtained from previous publications as described in the Materials and Methods (hESCs, hESC-NSCs, hESC-SCNTs). Data are average values of 3 independent experiments.

Using microarray analysis, we further characterized HiPSCs and HiPSC-derived cells. A heatmap illustration of microarray analyses (Fig. 5A), showed that the levels of expression of embryonic gene profiles in HiPSCs are similar to human embryonic stem cells (hESCs). These embryonic genes were mostly absent in the differentiated cell types including neuronal stem cells derived from hESCs (hESC-NSCs), HFNs, HiPSC-Ns as well as in HUVECs. It is noteworthy that although we selected the genes that are reported to be involved in embryonic development, not all these genes are necessarily exclusive for pluripotency. For example, FGF4 expression by endothelial cells has been previously reported [34]. In addition, a heatmap illustration of endothelial specific genes demonstrated that these genes are absent/minimal in HiPSCs, and other non-endothelial cells (Fig. 5B), confirming that HiPSCs are indeed pluripotent cells.

We further characterized the expression of various lineage markers, including neuronal and glial genes, in the differentiated HiPSC-Ns in comparison with HFNs and hESC-NSCs. Consistent with our immunoflourescent analysis, HiPSC-Ns expressed neuronal and glial genes, and clustered with HFNs and hESC-NSCs. However, expression levels of various neuronal markers were lower in HiPSC-Ns than HFNs and hESC-NSCs, indicating that there are more matured neurons and glial cells in HFNs whereas a fraction of pluripotent cells still remained undifferentiated in HiPSC-Ns (Fig. 5C, and see S1 Fig.). Since this neuronal differentiation protocol was developed by modifications of a cardiomyocyte differentiation protocol [21], we analysed the microarray data for the expression of cardiomyocyte genes in HiPSC-Ns. The analysis indicated no increase in expression of cardiomyocyte genes (S1 Fig.). Lack of potential cardiomyocyte as well as lack of endothelial specific gene expression strongly suggests that in addition to the cells belonging to neuronal lineage, the other major component of HiPSC-Ns are undifferentiated HiPSCs (S1 Fig.).

To further characterize the population of HiPSC-derived cells, we used flow cytometry and we probed for Sox2 (to identify embryonic and neural stem cells), CD44 (to identify glial cells and astrocyte precursors), and GFAP that is a marker for mature astrocytes. The results indicated that both HFNs and HiPSCs contain both mature and precursor glial cells as well as embryonic and neural stem cells. However, the amounts of mature astrocytes are lower in HiPSCs compared to HFNs (S2 Fig.).

### HiPSC-derived neurons possess similar morphology but longer neuritic processes compared to HFNs

**Fig 6:**
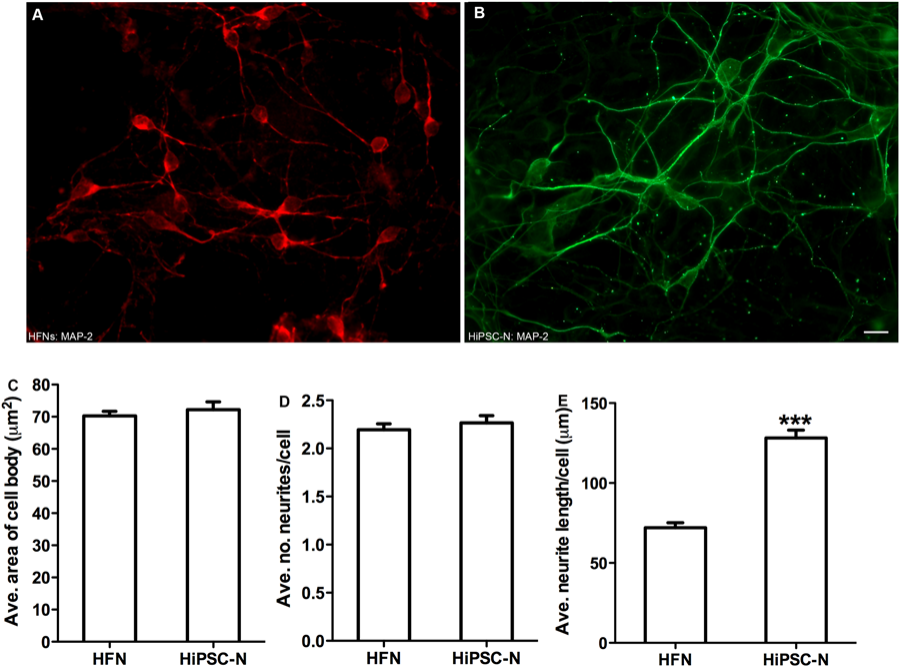
Morphological comparison of human fetal neurons (HFNs) and HiPSC-derived neurons. Neurons were immuno-stained for MAP-2 and their morphology was manually evaluated. Micrographs showing MAP-2 positive (A) HFNs and (B) HiPSC-derived neurons. The graphs compare average (C) size of cell bodies, (D) number of neurites/cell, and (E) neurite length/cell of HFNs and HiPSC-derived neurons. P< 0.001; Scale bar: 100 μm. The experiment was repeated 3 times, and at least 50 neuronal cells were evaluated in each group.

The morphology of neurons is critically relevant in forming neuronal networks and synapses [35–38]. Therefore in this study, we compared the morphology of HiPSC-derived neurons with the established HFNs. To minimize physical overlapping of neurons or neuronal processes, cells were cultured at approximately 20% lower than the routine density. Neurons were immuno-stained for βIII-tubulin/MAP-2 and their morphology (area of the cell body, number and length of neuronal outgrowths/cell) was manually evaluated. Morphological evaluations revealed that HiPSC-derived neurons have similar average size of cell body (approximately 70 μm^2^) and number of neurites/cell (approximately 2.3) when compared to primary human fetal neurons (Fig. 6A, B, C and D). Nevertheless, HiPSC-Ns possess significantly longer neuritic processes (approximately 140 μm) in comparison to HFNs (approximately 70 μm) (Fig. 6E.; P<0.001). We speculate that neurons possessing long neuritic outgrowths may have an advantage, at least *in vitro*, in forming an increased number of synapses with subsequent efficient inter/intra-neuron transfer of information.

### HiPSC-derived neurons are susceptible to inflammatory cell-mediated neuronal injury

**Fig 7:**
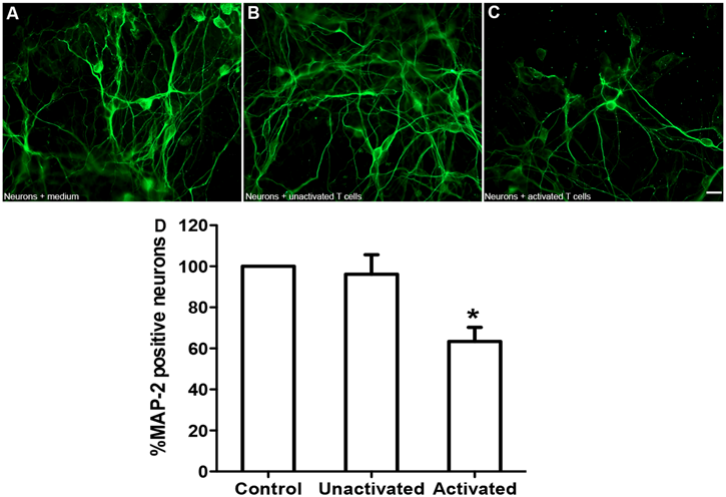
Susceptibility of HiPSC-derived neurons to inflammatory cells. MAP-2 immuno-stained micrographs showing that HiPSC-derived neurons were either (A) treated with neuronal media, (B) co-cultured with unactivated T cells or (C) co-cultured with activated T cells. (D) shows quantification of MAP-2 positive surviving neurons.

It is widely accepted that inflammation plays a major role in the pathogenesis of neurodegenerative diseases including MS [39], Alzheimer’s disease [40,41] and Parkinson’s disease [42]. We have previously used HFNs and T-cells as a model to mimic inflammation-mediated neurodegeneration *in vitro* [26,33]. Therefore, in these experiments, we evaluated the susceptibility of these newly generated HiPSC-derived neurons to lymphocyte cytotoxicity. For these studies, human PBMCs were activated using anti CD3 antibody in vitro for 3 days. Then, in a 1:1 ratio, the activated T cells were co-cultured with the HiPSC-derived neurons. The control groups were either treated with only media (without T cells) or co-cultured with unactivated T cells. To evaluate the cytotoxic effect of T cells on neurons, more than 12 fields per well/cover slip were randomly and manually counted consistently along the diameter of the cover slips using 40X objective fluorescence microscopy. The mean value of the control neuronal culture, which was not exposed to T cells, was normalized as 100%. The average number of MAP-2 positive neurons/group was inferred as a percentage of the control neuronal culture group. MAP-2 stains the cytoskeletal microtubules of the neurons including dendrites and axons. It was chosen because the disappearance of MAP-2 immunoreactivity is associated with neuronal injury and death in both *in vivo* and *in vitro* approaches [26,43–47]. Immuno-staining using MAP-2 showed that the density of the HiPSC-neurons when co-cultured with activated T cells was significantly reduced, whereas the density of the neurons in the control groups was unchanged (Fig. 7A, B and C). Quantification of these neuronal cultures shows that, unlike the control groups, activated T cells induced approximately 40% neuronal cells loss within 24 h (Fig. 7D; P<0.05). These data demonstrate that HiPSC-derived neurons are susceptible to inflammatory cells and may serve as a cell model in studies of inflammation-mediated neurodegenerative diseases.

P < 0.05. Scale bar: 200 μm. The experiment was repeated at least 3 times; and at least 3 wells were quantified from each condition.

### Relevant amino acids in HiPSC-derived neurons

**Fig 8:**
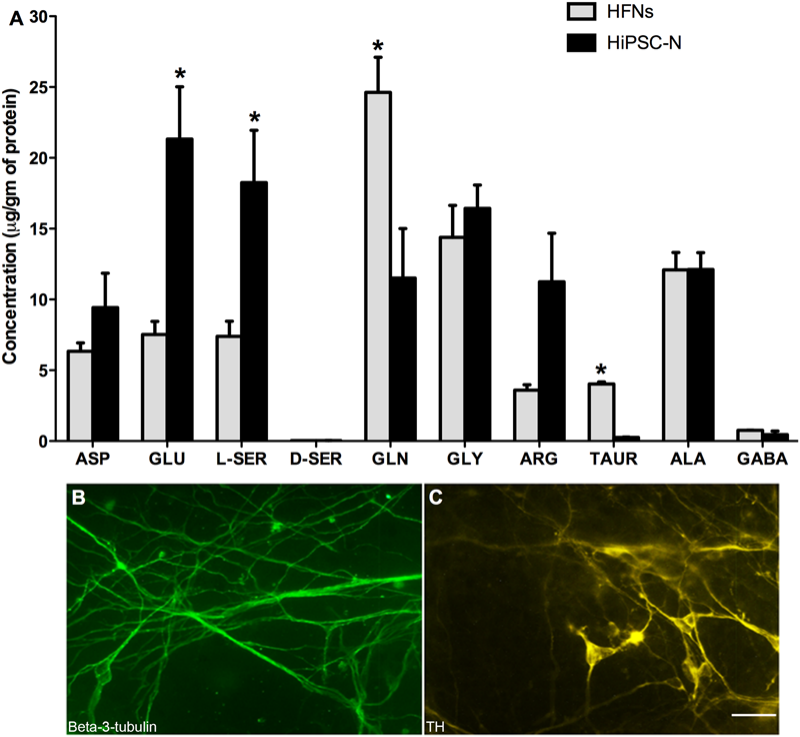
Levels of amino acids and expression of neuronal markers. (A) HPLC results showing the levels of amino acids in both HFNs and HiPSC-derived neurons. ASP = Aspartate, GLU = glutamate, SER = Serine, GLN = glutamine, GLY = Glycine, ARG = Arginine, TAUR = Taruine, ALA = Alanine, GABA = gamma-aminobutyric acid. Data are pooled from 3 independent experiments. (B) βIII-tubulin immuno-stained total HiPSC-derived neurons. (C) Tyrosine hydroxylase (TH)-positive dopaminergic neurons. Scale bar: 200 μm.

Amino acids play important roles in CNS function, including acting as neurotransmitters/neuromodulators in some cases (e.g. glutamate and GABA), and can influence behaviors under physiological conditions. Neurological and psychiatric disorders are often related to changes in the brain levels of amino acids, and precise quantification and profiling of amino acids demands high-performance methods [48,49]. With the objective of screening the profiles and levels of several important bioactive amino acids, some of which are related metabolically and/or functionally to glutamate and/or GABA, we used HPLC with fluorescence detection and assessed the types and amounts of these amino acids in HiPSC-derived neurons in comparison to primary HFNs. To carry out this objective, both cell types were cultured and their respective proteins were isolated. The levels of 10 amino acids essential to CNS function were measured by HPLC. HiPSC-derived neurons contained almost all of these amino acids. Both HiPSC-derived neurons and HFNs had similar levels of aspartate, glycine, arginine and alanine; D-serine was absent, and the levels of GABA were very low in both cell types. Amino acid composition of HiPSC-derived neurons was dominated by glutamate and L-serine, which were present at significantly higher levels in HiPSC-neurons than in HFNs, whereas glutamine was dominant and present at a substantially higher level in HFNs (P<0.05). The levels of taurine were low in both cell types but significantly lower in HiPSC-neurons (Fig. 8A; P<0.05).

Dopaminergic neurons are an essential model for studying the pathogenesis of Parkinson’s disease. Hence, within the βIII-tubulin positive HiPSC-derived neuronal population (Fig. 8B), we assessed the presence of dopaminergic neurons. Using anti-tyrosine hydroxylase (TH) antibody, some dopaminergic neurons were detected in HiPSC-neuronal cultures (Fig. 8C).

### HiPSC-derived neurons and astrocytes express functional neurotransmitter receptors

**Fig 9:**
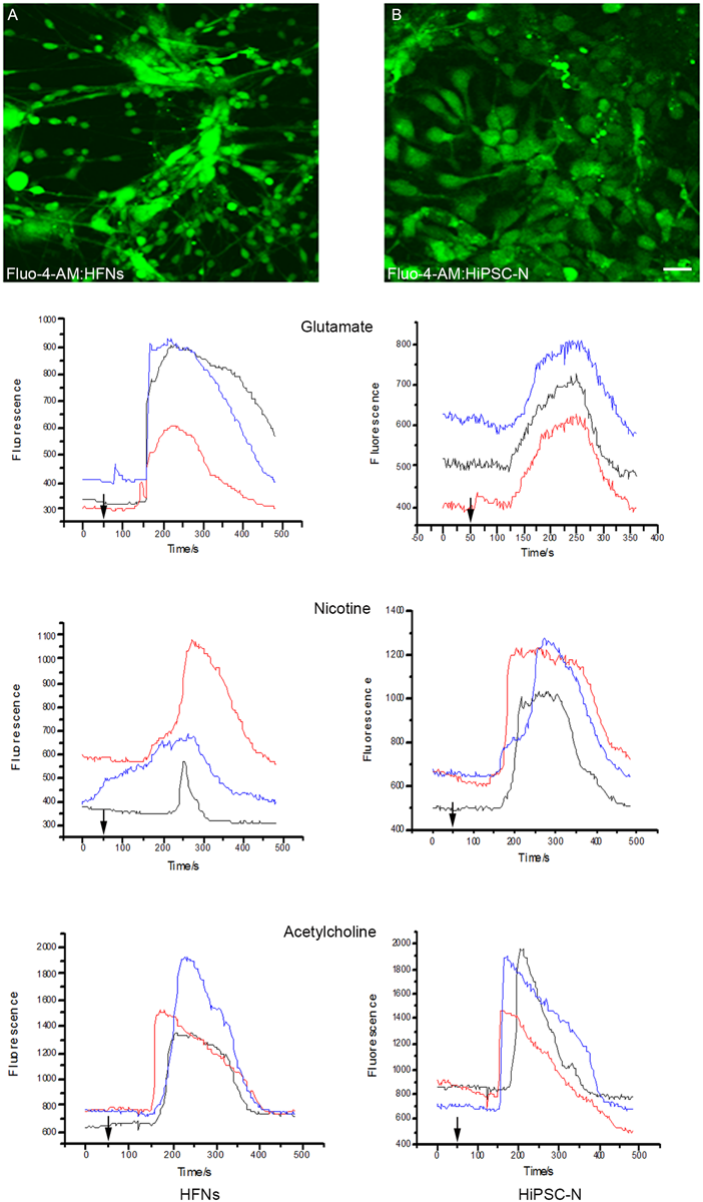
Calcium imaging and expression of functional receptors on neurons. (A) Primary human fetal neurons (HFNs) and (B) HiPSC-derived neurons (HiPSC-Ns) were incubated with the membrane-permeant acetoxymethyl form of the fluorescent Ca^2+^-sensitive dye Fluo-4-AM for 1hr prior to imaging, after which the fluorescence intensity was measured. Graphs shown on left column represent responses of three HFNs, and on right responses of three HiPSC-Ns to glutamate, nicotine and acetylcholine treatments. Arrows indicate the time when the stimulants were applied. Scale bar: 100 μm. The experiments were repeated 3 times.

**Fig 10:**
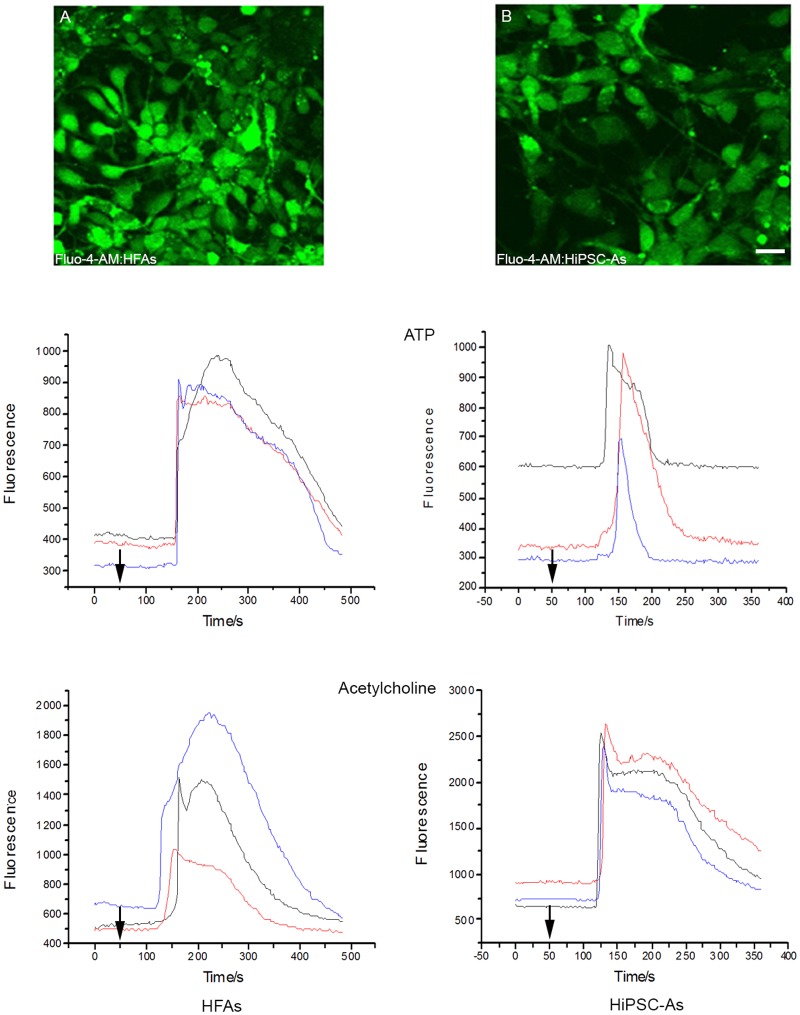
Calcium imaging and expression of functional receptors on astrocytes. (A) Primary human fetal astrocytes (HFAs) and (B) HiPSC-derived astrocytes (HiPSC-As) were incubated with the membrane-permeant acetoxymethyl form of the fluorescent Ca^2+^-sensitive dye Fluo-4-AM for 1hr prior to imaging, after which fluorescence intensity was measured. Graphs shown on left column represent responses of three HFAs and on right responses of three HiPSC-As to ATP and acetylcholine treatments. Arrows indicate the time when the stimulants were applied. Scale bar: 100 μm. The experiments were repeated 3 times.

Calcium imaging in HiPSC-derived neurons and astrocytes was employed to measure and monitor the dynamic changes of the free cytosolic Ca^2+^ concentration in response to neurotransmitters and other stimulants. The selection process/criteria for a neuron or an astrocyte in a culture containing both cell types, whether HiPSC-derived or primary human fetal cells (Fig. 9A and B, Fig. 10A and B), was based on apparent morphological variations between astrocytes and neurons (astrocytes are bigger and morphologically different from neurons), as well as on immunocytochemical characterization using neuronal and astrocyte markers, as shown in Fig. 4. Both the established HFNs and HiPSC-derived neurons instantly responded to treatment with the stimulants (glutamate, nicotine and acetylcholine) by elevating their Ca^2+^ concentrations. However, the magnitude of the average peak values of glutamate-mediated responses was significantly higher in primary HFNs than in HiPSC-derived neurons. On the contrary, the response to nicotine was substantially higher in HiPSC-neurons compared to HFNs. Acetylcholine treatment showed similar responses in both cell types (Fig. 9A, HFNs vs. B, HiPSC-derived neurons; and see table 2 for the actual values). Similarly, acetylcholine and ATP applications increased calcium signals in primary human astrocytes as well as in HiPSC-derived astrocytes. However, the mean values of responses to acetylcholine and ATP were significantly higher in HiPSC-derived astrocytes compared to primary human fetal astrocytes (HFAs) (Fig. 10A, HFAs vs B, HiPSC-derived astrocytes; and see table 2).

**Table 2:** Summary of cellular response to stimulants.

Treatment		HFN	HiPSC-N	HFA	HiPSC-A
Glutamate	Mean peak value	450 ± 76.4	217.3 ± 3.5		
	# of cells analyzed	14	15		
	# of replicates	3	3		
Nicotine	Mean peak value	330.3 ± 88.3	589.3 ± 28.8		
	# of cells analyzed	14	15		
	# of replicates	3	3		
Acetylcholine	Mean peak value	1032 ± 212.7	1041.3 ± 129.5	681.3 ± 126.9	1764 ± 68.0
	# of cells analyzed	14	15	15	15
	# of replicates	3	3	3	3
ATP	Mean peak value			540.7 ± 36.4	800 ± 132.3
	# of cells analyzed			15	15
	# of replicates			3	3

Summary table showing the treatment of primary human fetal neurons (HFNs), HiPSC-derived neurons (HiPSC-Ns), primary human fetal astrocytes (HFAs), and HiPSC-derived astrocytes (HiPSC-As) with different stimulants (glutamate, nicotine and acetylcholine for neurons, and ATP and acetylcholine for astrocytes), and the responses of the cells to each treatment. The average peak value of the cell responses to each stimulant treatment is described as mean ± SEM of fluorescent intensity. The number of cells analyzed for each treatment in each group of cells and the number of replicates for each experiment are indicated.

All the experiments described were performed at least 3 times. On average 14–15 cells were recorded in each experiment. The mean peak values of cells’ responses to the treatments with the stimulants, the number of cells assessed in each application and the number of replications of the experiments are summarized in Table 2. Altogether, these results suggest that HiPSC-derived neurons and astrocytes are physiologically as functional as primary human fetal cell-derived neurons and astrocytes.

## Discussion

The late stages of neurodegenerative diseases are characterized by massive neuronal death, and therapeutic interventions late in the course of the disease remain ineffective [13]. The mechanisms of these conditions are difficult to study because of inaccessibility of human tissues [11] and lack of reliable animal models that accurately recapitulate the pathogenesis of human neurodegenerative diseases, thus impeding the development of effective drugs [4]. The advent of iPSC technology where differentiated somatic cells are induced to pluripotency state by ectopic expression of defined factors [8] has provided an unprecedented opportunity to use readily available and physiologically relevant human cells as *in vitro* models. iPSCs have nearly identical genetic and functional properties to human ESCs [50]. The recent progress in site-specific gene editing technologies using zinc-finger nucleases and transcription activator-like effector nucleases has advanced the feasibility of gene correction in patient-specific iPSC lines [18,19,51]. Hence, patient-specific and disease-specific iPS cell lines, and their derivative cells, are being generated from various somatic cell sources to model different human diseases *in vitro* [11,52].

However, the choice of the source of somatic cells is critical to the generation of clinically relevant iPS cell lines and their derived cells. Based on their accessibility without invasive methods, expansion capacity, efficient reprogramming, and minimal *in vitro* manipulations, HUVECs present an optimal somatic cell source for iPSC generation [16]. Considering that umbilical cord-derived endothelial cells are coming from a newborn, they may have acquired fewer genetic mutations compared to adult-derived somatic cells [4,16]. These criteria make HUVECs a highly attractive source for generation of iPSCs and subsequent derivation of relevant cell types for use in studies of diseases with genetic components.

Appropriate control for patient-derived iPSCs is a crucial issue. Using ESCs or iPSCs from other genetically unrelated healthy donors as controls and comparing them with patient derived cells could be a source of controversy because of differences in genetic backgrounds of the two populations. iPSCs generated from cells of healthy family members are better controls as they may possess fewer genetic variations compared to patient-derived iPSCs [18,19]. For example, in a study conducted on MS patients, the authors suggested using a more homogenous genetic pool of cases and controls in order to have the most significant MS-associated single-nucleotide polymorphism [53]. The incidence for first-degree relatives of MS patients is 2% to 5%, unlike in the general population where it is less than 0.1% [54]. The relevance and benefits of early detection or genetic predisposition prediction in familial related neurodegenerative diseases reviewed by Paulsen et al. [1] make HUVECs an attractive source of iPSCs and derivative cells to model neurodegenerative diseases. Moreover, among newborns at risk to carry a disease-causing genetic mutation, planned harvest of fetal HUVECs and generation of HiPSC-N cells will facilitate diagnostic testing, and perhaps patient-specific drug- or cell-based therapies.

In this study, we generated iPS cell lines from HUVECs in accordance with earlier reports [14–16]. The 4 defined transcription factors (Sox2, Oct3/4, Klf4 and c-Myc) [9] or (Sox2, LIN28, Oct4 and Nanog) [55] are used in most studies. Others generated iPSCs using 6 factors (Sox2, Oct3/4, Klf4, LIN28, Nanog and c-Myc) [12]. HUVEC-derived iPSCs can also be generated using only two factors (e.g., Sox2 and Oct4); however, the use of fewer factors likely contributes to the lower reprogramming efficiencies and delayed reprogramming kinetics [17,56]. Hence, we used 6 factors in our study; and demonstrated that HUVECs acquired ESC-like morphology and expressed embryonic genes but lost endothelial cell identities during reprogramming, which is a hallmark of induced pluripotency. We next developed a novel protocol that involves treatment with bone morphogenetic proteins (BMP4) and Activin A, which are members of the transforming growth factor β (TGFβ) superfamily, to generate neuronal and astrocytic cells from HUVEC-derived iPSCs and proceeded to determine their functional properties. Although Activin A is considered a negative regulator of neuron differentiation [57], many studies have reported that TGFβ superfamily members are important regulators of neuronal lineage induction and survival [58–63]. Neuronal differentiation was induced by blocking both the mesodermal and endodermal lineages through the use of dual SMAD inhibition, and these inhibitions act on the downstream molecules of the Activin/ TGFβ and BMP signaling pathways [64]. BMPs regulate cell fate decisions in stem cells [65]. Activin A and BMP4, in the presence of B27, have been used to generate cardiomyocytes from human embryonic stem cells [21]. B27 growth factor was originally identified as a growth factor for neurons [66]. In addition, neuronal and cardiac differentiation occurs in parallel, and this concurrent development of cardiomyocytes and neurons suggests bidirectional communication between both cell types [67]. Based on these reports, we hypothesized that treatment of HiPSCs with Activin A, BMP4 and B27 may lead to differentiation of HiPSCs towards neuronal cell fate. Our protocol demonstrated successful direct differentiation of HiPSCs into neuronal phenotype and, furthermore, similar to primary human fetal cells which differentiate into neurons and glial cells, HiPSCs also generated both astrocytes and neurons. Astrocytes play critical roles in promoting morphological and functional maturation of HiPSC-derived neurons [68]. Human neurons cultured on astrocytes for more than 2 months continue their developmental process by showing synchronized network activity [69]. This simultaneous generation of neurons and glial cells is relevant in disease modeling.

Neurodegeneration is a slow process that begins decades before the onset of the actual symptoms of the disease [70]. Nevertheless, in some of familial forms of diseases such as Alzheimer’s disease, abnormalities have been noticed as early as developmental stage [71]. HiPSCs as well as the derived neurons and astrocytes offer new opportunities to study these early defects and the mechanisms of disease progression. We compared the morphology of HiPSC-derived neurons with the established primary HFNs. The size of the cell body and average number of neurites per cell of HiPSC-derived neurons and HFNs are almost identical. However, the neurite lengths of HiPSC-derived neurons were significantly longer compared to HFNs, and this might be related to higher heterogeneity of the HiPSC-derived neurons compared to HFNs, which are mainly represented by cortical neurons. In addition, the complexity of morphological features and dendritic arborization is an important indicator of neuronal maturation [68] and it produces more neuronal connectivity and efficient transfer of information inter-and intra-neuron [35–38,72]. Moreover, HiPSC-derived neurons are susceptible to inflammatory cells, making them a good model to study inflammation-mediated injury, a process that plays an important role in the pathogenesis of many neurodegenerative diseases [39,40,42].

Neurological and psychiatric disorders are often influenced by the levels of amino acids such as glutamate and GABA, which are important neurotransmitters in the CNS [48,49]. Upon differentiation, HiPSC-derived neurons expressed various amino acids at similar or higher levels compared to HFNs. D-Serine was absent in both cell types whereas taurine and gamma-amino-butyric acid (GABA) were low. The domination of glutamate in HiPSC-derived neurons can be related to the differences in the differentiation stages of these cells. Indeed, it has been suggested that glutamatergic neurons are generated before GABAergic neurons during the differentiation process of HiPSCs into neurons [68]. In our experiments, HiPSCs were cultured for maximum of a couple of weeks and this short time frame may not allow adequate differentiation of GABAergic neurons. However, some of the βIII-tubulin positive neurons were dopaminergic neurons, which highlights the potential relevance of these HiPSCs in the study of Parkinson’s disease.

A major factor in determining the feasibility and appropriateness of these iPSC-derived neurons as tools for future exploration of the pathogenesis of target CNS diseases was to assess whether HiPSC-derived neurons and astrocytes are functional. Ca^2+^ channels have major roles in regulating intracellular Ca^2+^ homeostasis and neuronal excitability [73]. HiPSC-derived neurons and astrocytes respond to variety of stimulants such as nicotine, ATP, glutamate and acetylcholine. Glutamate is the most common mammalian CNS neurotransmitter mediating chemical signaling inter- and intra-neuronally and from neurons to astrocytes; and ATP is the main transmitter molecule implicated in astrocytic signaling [74,75]. We showed that the intracellular concentrations of calcium in HiPSC-derived neurons strongly respond to glutamate, nicotine and acetylcholine treatments, to a similar or higher level than HFNs. This suggests that the molecular and physiological features of these receptors are similar to those of primary CNS neurons. Similarly, ATP and acetylcholine treatments evoked high signals in both HiPSC-derived astrocytes as well as in primary human astrocytes, and this might indicate the functional similarity of the two cell types.

In conclusion, HUVECs are an ideal cell source for the generation of therapeutic-grade iPSCs and the derivative cells. Hence, we have successfully reprogrammed HUVECs into HiPSCs, and established a novel and direct-differentiation protocol without the use of feeder cells or EB, and generated HiPSC-derived neurons and astrocytes with a high efficiency. The morphological, chemical and physiological properties of HiPSC-derived neurons and astrocytes indicate the cells have comparable features with primary human fetal neurons and astrocytes. Our data suggest that HiPSC-derived neurons and astrocytes will offer unlimited cell resources with which to model and study the pathogenesis and progression of various diseases of the CNS, as well as to facilitate development of new drug- and cell-based therapeutic strategies.

## Supporting Information

S1 Fig Microarray-based gene analysis of HUVECs and HiPSC- derived neurons. Transcript set score showing the expression levels of pluripotent, cardiac, endothelial and neuronal genes by HUVECs, HiPSCs, HiPSC-derived neurons (HiPSC-Ns), HFNs and samples obtained from previous publications as explained in the Materials and Methods (hESCs, hESC-NSCs, hESC-SCNTs).
(TIF)

S2 Fig FACS analysis showing Sox2, CD44 and GFAP- positive cells in human fetal brain-derived cells (upper two rows) and HiPSC-derived cells (lower two rows). (TIF)
