# Supplementary figures and images for: Reprogramming of HUVECs into Induced Pluripotent Stem Cells (HiPSCs), Generation and Characterization of HiPSC-Derived Neurons and Astrocytes
Source: PLoS One. 2015 Mar 19;10(3):e0119617. doi: 10.1371/journal.pone.0119617 (PMC4366250; doi:10.1371/journal.pone.0119617)

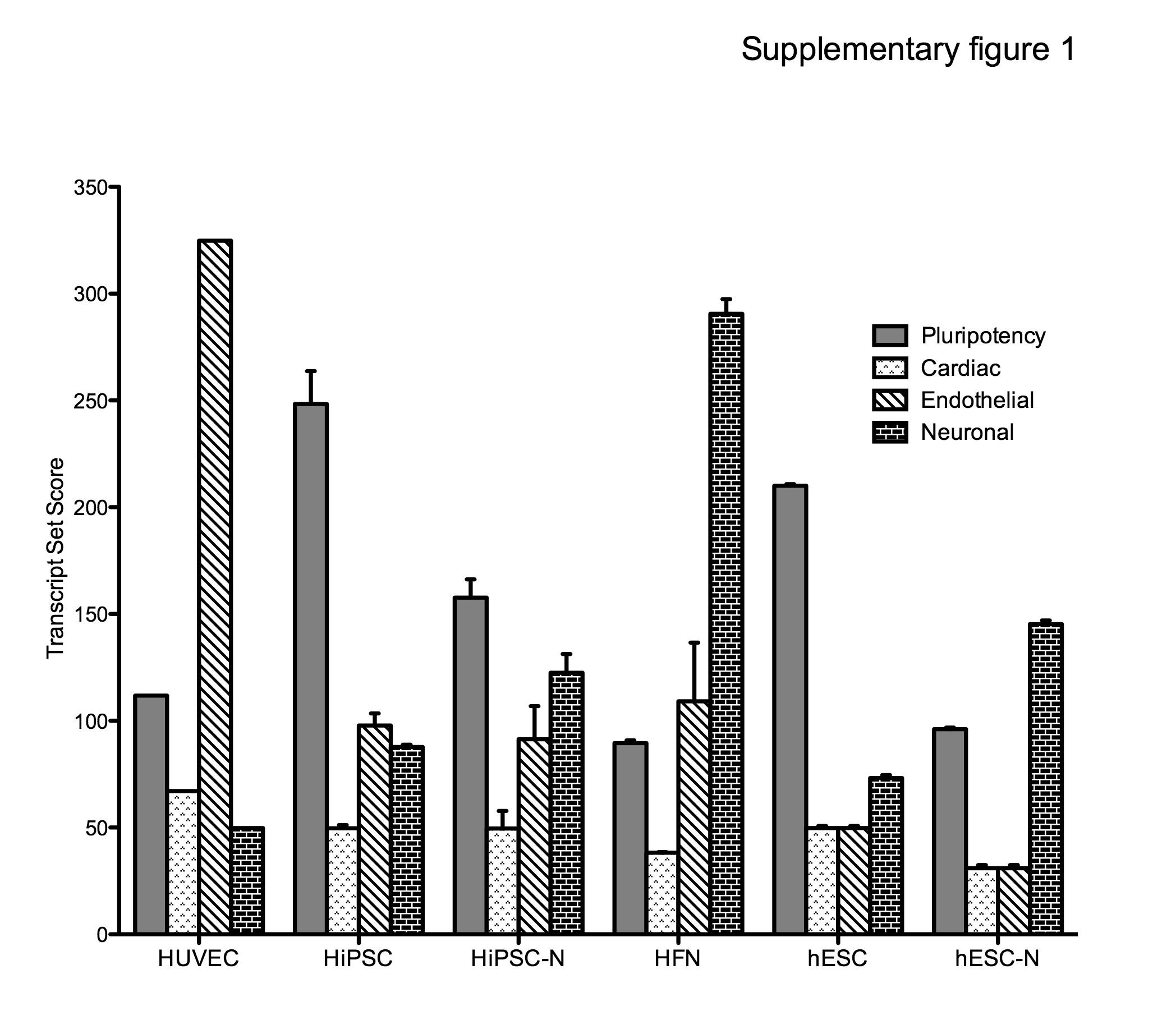

Supplement: S1 Fig — Transcript set score showing the expression levels of pluripotent, cardiac, endothelial and neuronal genes by HUVECs, HiPSCs, HiPSC-derived neurons (HiPSC-Ns), HFNs and samples obtained from previous publications as explained in the Materials and Methods (hESCs, hESC-NSCs, hESC-SCNTs). (TIF) [file pone.0119617.s001.tif]

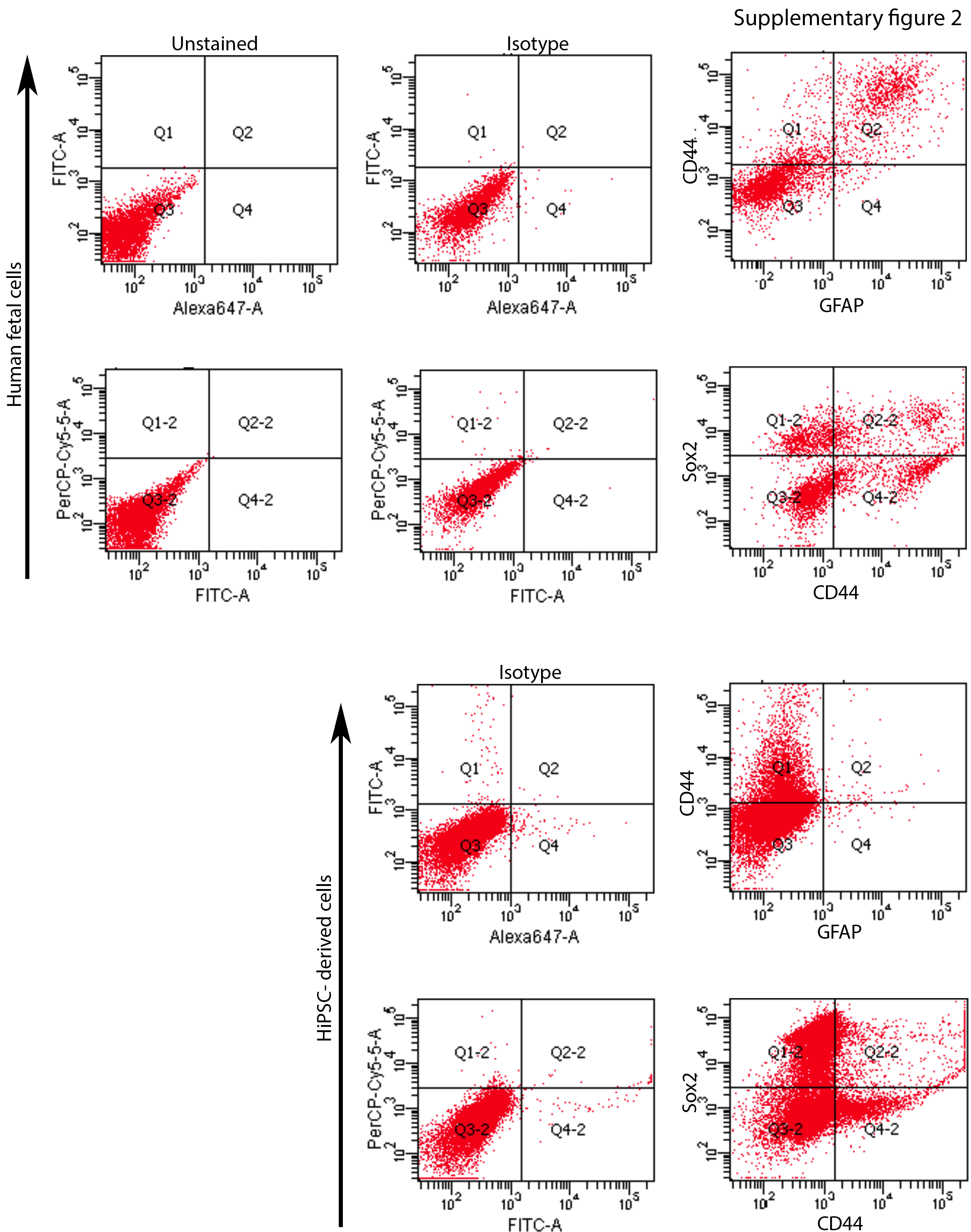

Supplement: S2 Fig — (TIF) [file pone.0119617.s002.tif]
